# Mask-wearing selectivity alters observers’ face perception

**DOI:** 10.1186/s41235-022-00444-z

**Published:** 2022-11-16

**Authors:** Erez Freud, Daniela Di Giammarino, Carmel Camilleri

**Affiliations:** Department of Psychology and Centre for Vision Research, York University, Toronto, Canada

**Keywords:** Face perception, Masks, Embodied cognition

## Abstract

Face masks became prevalent across the globe as an efficient tool to stop the spread of COVID-19. A host of studies already demonstrated that masks lead to changes in facial identification and emotional expression processing. These changes were documented across ages and were consistent even with the increased exposure to masked faces. Notably, mask-wearing also changes the state of the observers in regard to their own bodies and other agents. Previous research has already demonstrated a plausible association between observers’ states and their perceptual behaviors. Thus, an outstanding question is whether mask-wearing would alter face recognition abilities. To address this question, we conducted a set of experiments in which participants were asked to recognize non-masked faces (Experiment 1), masked faces (Experiment 2) and novel objects (Experiment 3) while they were either masked or unmasked. Mask wearing hindered face perception abilities but did not modulate object recognition ability. Finally, we demonstrated that the decrement in face perception ability relied on wearing the mask on distinctive facial features (Experiment 4). Together, these findings reveal a novel effect of mask-wearing on face recognition. We discuss these results considering the plausible effect of somatosensory stimulation on visual processing as well as the effect of involuntary perspective taking.

## Significant statement

During the COVID-19 pandemic, the use of face masks became prevalent across countries and societies in an effort to curb virus transmission. A host of studies already demonstrated that masks lead to changes in facial identification and emotional expression processing. Notably, an unexplored aspect of this phenomenon is whether mask-wearing (i.e., when the observers wear a mask) also changes how we perceive faces. This question pertains to more general research domains with implications for everyday life: How does the state of an observer, in relation to their own body and other individuals, modulate their perceptual behaviors? In a series of experiments, we demonstrate that mask-wearing hinders observers’ ability to recognize masked and non-masked faces. This effect is specific to face recognition, and evident only when observers wear a mask that occlude distinctive facial features. These results provide timely insights into the effect of mask-wearing on visual perception and also reveal non-visual processes that modulate the way we perceive the world around us.

## Introduction

The onset of the COVID-19 pandemic saw an unprecedented rise in the use of face masks among members of the general public as governments around the world mandated mask-wearing in public spaces (Canada, 2020). This new constraint was introduced in an effort to curb virus transmission and allow for the safe re-opening of educational, economical and recreational institutions. Despite playing a crucial role in facilitating a return to normalcy, masks have propelled society into a new arena of facial recognition, one in which familiar and unfamiliar faces alike are now partially obscured from view. Consistently, a great deal of research has been dedicated to estimating the effect of masks on face perception. Recent studies have found that masks pose a considerable challenge to face perception among adult populations, producing consistent deficits in face recognition abilities and leading to reduced holistic processing (Carragher & Hancock, 2020; Freud et al., 2020, 2022). Others have found that the occlusion of the lower face by a sanitary mask or scarf hinders our ability to interpret facial expressions (Calbi et al., 2021; Carbon & Serrano, 2021). Moreover, a recent study by Stajduhar and colleagues (2022) demonstrated that school-age children-much like adults-show a robust impairment in recognition of masked faces.

Research thus far has focused on what happens to observers’ face perception abilities when they are tasked with identifying others who wear a mask. However, it is essential to also consider the complementary situation: what happens to face perception abilities when the observers are the ones wearing the mask, particularly when the mask occludes distinctive face features that contribute to recognition? This question pertains to a more general research domain—to what extent do the state of the observers, in relation to their own body and to other agents modulate the way in which they process faces and other visual stimuli?

The effect of the observers’ bodily state on sensory processing was often explored by manipulating the body’s orientation relative to visual stimuli. In a previous study, body position was systematically manipulated while participants were asked to recognize letters and faces. The authors found a robust influence of body orientation on both categories, with a greater effect on face recognition (Barnett-Cowan et al., 2015). Along similar lines, superior face recognition performance was observed when participants were presented with egocentrically upright faces (i.e., faces that appear upright with respect to the observer’s body position) compared with environmentally upright faces (i.e., faces that appear upright with respect to the room they are in) (Davidenko & Flusberg, 2012). The effect of bodily states on fundamental perceptual behaviors was also demonstrated beyond face processing. For example, Kim and colleagues (2021) used virtual reality to show that body orientation affects the perceived size of visual targets, making objects appear smaller and closer when lying down. Together, these findings suggest that there is an association between body representations and our perception of the world around us, such that any modification to the former can affect our experience of the latter.

The above-mentioned association between face processing and bodily position was also established by studies in which emotional recognition was distorted when participants were blocked from imitating the expressions of those they were observing (Borgomaneri et al., 2020; Oberman et al., 2007). Along similar lines, reduced recognition of emotional states was also observed when TMS was applied to the right occipital face area (rOFA) but also to the face region of the right somatosensory cortex (rSC) (Pitcher et al., 2008; and also see Borgomaneri et al., 2015 for similar results during the perception of emotional body language). However, it is worth noting that most of these studies suggest that the involvement of somatosensory processing in face recognition selectively supports emotion recognition and not other aspects of face recognition (Adolphs et al., 2000; Sel et al., 2014).

Notably, in addition to modulation of the bodily state of the observers, wearing a mask also changes the state of the observer in relation to other agents. Namely, masked observers might consider that others cannot properly recognize them, which in turn can hinder their own face processing abilities. This idea is consistent with recent theories that claim that observers rapidly and involuntary consider the perspective of others when completing perceptual judgments (i.e., altercentric intrusions) (Kampis & Southgate, 2020; Samson et al., 2010). For example, the face-selective N170 ERP component is enhanced when viewing an inverted face. A similar enhancement was found when the observers were seated opposite to another person (i.e., the agent) who would view that face as inverted (upright for the observer, inverted for the agent), indicating that the observer also encodes the face from the others’ perspective (Böckler & Zwickel, 2013).

To conclude, face recognition is evidently not just a visual task and might be modulated by somatosensory stimulation that changes the observer’s bodily state as well as by the presence of other agents. Given that face masks cover important, distinctive features of the face (mouth and nose), we predicted that there would be a reduction in one’s recognition of faces when people wear masks and are tasked with recognizing other individuals. To test these predictions, we conducted a set of experiments in which non-masked (Experiment 1) and masked (Experiment 2) faces were presented while observers had the masks on or off. We also examined the specificity of the mask-wearing effect by testing object recognition abilities (Experiment 3) and by asking participants to wear the masks in an unconventional way such that important facial features would remain uncovered (Experiment 4).

## Experiment 1

### Methods

#### Participants

Given that our experiments were conducted online, we a-priori decided to collect data from a large sample for each experiment. Eighty participants (40 females, age: 27.3) were recruited online (https://www.prolific.co/) during the month of November 2021. Participants were compensated for their time (~ $3 CAD for 15 min). All experiments were conducted in accordance with the relevant guidelines and regulations of the ethics review board at York University. All participants provided informed consent prior to completing the experiment. Three participants were excluded from the analysis as their d’ scores were lower than 1. Additionally, data from participants (*n* = 11) who self-reported that they did not wear or remove their masks as instructed (see procedure) was excluded. Hence, the final sample included 66 participants. Notably, the reported results are fully replicable when all participants were included in the analysis. Data and analysis code for all experiments are available online on the Open Science Framework (https://doi.org/10.17605/OSF.IO/9A5J4).

#### Materials

**Fig. 1:**
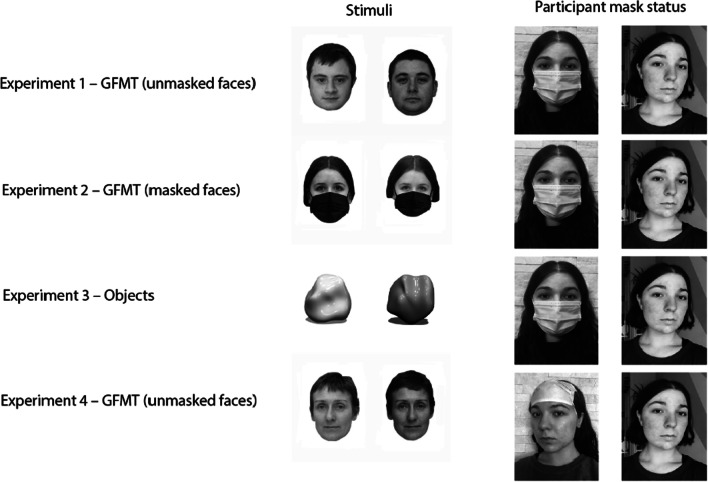
Experimental stimuli and condition. Examples of the stimuli used in each experiment (left panel), as well as the placement of participants’ masks throughout the experiment (right panel)

The extended version of the Glasgow Face Matching Test (GFMT: Burton et al., 2010) was used to assess face perception abilities. The task comprises 164 “same-or-different” two-alternative forced-choice decisions using paired images from a demographically heterogeneous sample of 300 people. Each face image is front facing in pose, neutral expression, and presented in black-and-white (see Fig. 1 for an example image pair and Burton et al. (2010) for further details) to subtend a visual angle of 6.5° × 4°. Given the online nature of the experiment, participants were asked to supply their own face masks. However, instructions specified that the mask should be either medical or surgical and be similar to those that participants would use during their daily lives.

#### Procedure

The experiment consisted of a same or different task whereby participants were presented with two faces either of the same person (different images) or two different persons. Participants then had to indicate—via a keypress—whether each pair of face photos depicted the same person (press “1”) or two different persons (press “2”). The task was self-paced and typically took about 10 min to complete. Participants were randomly assigned to one of two counterbalanced groups. The first group was instructed at the beginning of the experiment to retrieve a medical or surgical mask and wear it for half the experiment (84 trials), at which point they were given instructions asking them to remove their mask and continue. The second group completed the first portion of the experiment without wearing a mask and were then given similar instructions as the first group when they reached the halfway point. A questionnaire was included at the end of the experiment to gauge whether participants adhered to the instructions they were given. The questionnaire consisted of 11 questions on a 7-point Likert scale with responses ranging from strongly disagree (1) to strongly agree (7) (Table 1). Questions 1 and 3 were used to estimate the adherence with the experimental instructions. Question 2 and 5 were used to extrapolate information on participant’s experience with masks; the others served as distractors. Participants were informed that their compensation would not be affected by the truthfulness of their responses. Participants who self-reported that they did not wear or remove their masks as instructed (an average score of 5 or below for the two critical questions) were excluded from the final analysis.

**Table 1:** Questionnaire

Please select your answer to the following questions. Note that your compensation will not be affected by the truthfulness of your answers.
1. I wore my mask during the experiment as instructed
2. In my daily life, I need to wear a mask often
3. I did not take off my mask during the experiment until I was asked to
4. I have a lot of experience in encountering people who wear masks
5. I spend a lot of time in public places where mask wearing is common
6. Mask wearing is strongly enforced in my country of residence
7. Most people follow mask mandates in my country
8. I have difficulties recognizing familiar people (i.e., family members, colleagues, friends) when they are wearing masks
9. When people wear masks, I have difficulties in deciding whether they are familiar to me or not
10. Most people that I encounter everyday are wearing masks
11. In my daily life, I need to recognize a lot of people who wear masks

##### Data analysis

Data was processed using Python and statistical analyses were conducted using JASP (JASP team, 2022). Sensitivity scores (*d*′) and reaction times served as the two dependent variables. Sensitivity (*d*′) scores were calculated for each participant using the following formula: *d*′ = *z*(hit) − *z*(false alarms). To compare mask versus no-mask conditions, we employed Bayesian pairwise *t* tests.

## Results

The aim of Experiment 1 was to assess the extent to which wearing a mask interferes with face recognition processes. To this end, participants completed the extended version of the GFMT (Burton et al., 2010) while they were either wearing or not wearing a mask.

**Fig. 2:**
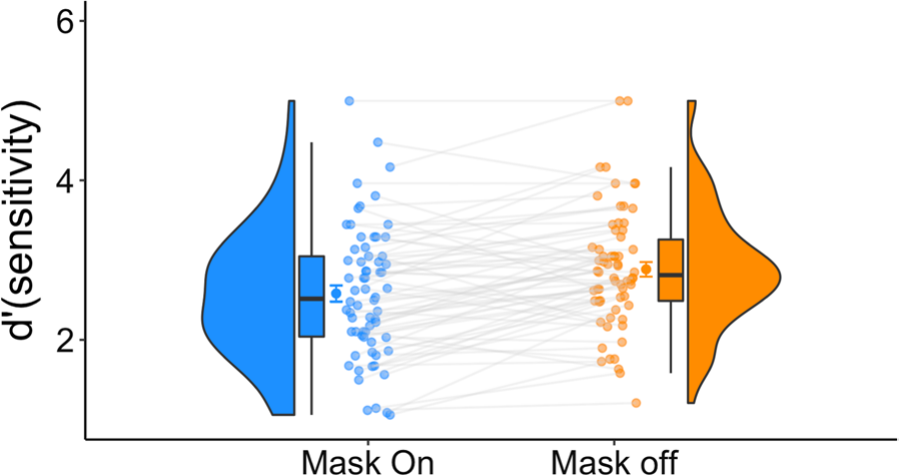
Experiment 1 results. Better accuracy results were observed for the mask-off condition. For each condition, we present the distribution, the individual data points, and the quartiles of the dataset. The whiskers depict the rest of the distribution excluding outliers. The darker dots denote the means, and the error bars denote one standard error

We found that observers were more accurate in the GFMT when they did not wear their masks (Fig. 2). The Bayesian *t* test robustly confirmed the difference between the two conditions [${\text{BF}}_{10}$ = 609], suggesting that the data are approximately 609 times more likely to occur under $H_{1}$ than under $H_{0}$. Reaction times were similar across the two conditions [BF_10_ = 0.1145]. Thus, our results provide novel evidence that mask wearing can hinder face perception abilities.

### Experiment 2

The results of Experiment 1 demonstrated that mask wearing can hinder face recognition. However, an alternative explanation for these results is that the decreased performance for masked faces reflects the incongruency between the observer state (masked) and the stimuli (non-masked). To disentangle these two alternative accounts, we repeated Experiment 1, but utilized a masked version of the GFMT. If the observer masking status modulates face recognition abilities, the results of Experiment 1 should be replicated. In contrast, if the observer-stimulus incongruency mediated the results of Experiment 1, we expected to find a reversed pattern such that participants would exhibit superior performance while wearing a mask.

### Methods

#### Participants

Eighty participants (40 females, mean age: 24.8) were recruited online (https://www.prolific.co/) during the month of December 2021, none of whom participated in the previous experiment. Participants were once again compensated for their time (~ $3 CAD for 15 min). Six participants were excluded from the analysis as their *d*′ scores were lower than 1. Additionally, data from participants (*n* = 8) who self-reported that they did not wear or remove their masks as instructed was excluded. Hence, the final sample included 66 participants. The reported results are fully replicable when all participants were included in the analysis.

#### Materials and procedure

An edited version of the extended Glasgow Face Matching Test (GFMT) was used to assess face perception abilities in which identical face masks were superficially added onto each pair of faces using photoshop (See Fig. 1 for an example image pair). Participants were once again asked to supply their own medical or surgical face mask to wear during select parts of the experiment.

## Results

**Fig. 3:**
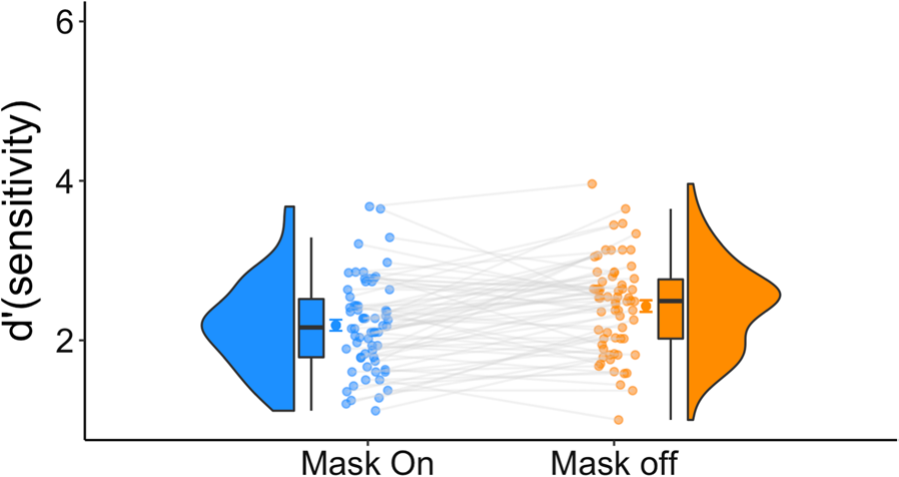
Experiment 2 results. Better accuracy results were observed for the mask-off condition. For each condition, we present the distribution, the individual data points, and the quartiles of the dataset. The whiskers depict the rest of the distribution excluding outliers. The darker dots denote the means, and the error bars denote one standard error

Experiment 2 explored the extent to which wearing a mask interferes with the observers’ ability to recognize other masked faces. To this end, participants completed an edited version of the GFMT in which the face stimuli were all masked. We once again conducted a Bayesian *t* test to compare the two mask status conditions and found robust evidence in favor of $H_{1}$ [${\text{BF}}_{10}$ = 29; the observed data are about 29 times more likely under *H*_1_], such that superior performance was found for the non-masked condition (Fig. 3). Similar to Experiment 1, no effect of RT was observed [${\text{BF}}_{10}$ = 0.138; the observed data are about 7 times more likely under *H*_0_]. These results refute the notion that the observer-stimulus incongruency mediated the effect observed in Experiment 1 and suggest that wearing a mask hinders face recognition abilities.

Next, we sought to explore whether experience with mask-wearing, prior to or throughout the experiment modulated the observed mask effect. To this end, we first summed the scores of items 2 and 5 of the questionnaire as an indicator for mask-wearing experience. We then correlated the experience score with the mask wearing effect (i.e., *d*′ non-masked − *d*′ masked). Across the two experiments, we did not find evidence for a person correlation between the two variables (Exp 1: *r* = 0.089, *p* = 0.47; Exp 2: *r* = − 0.21, *p* = 0.0.078), weakening the hypothesis that mask-wearing experience attenuates the effect. Next, we also examined whether the mask-wearing effect was modulated as a function of short-term experience. We split each experimental block into two (42 trials each) and examined whether the mask effect was more evident in the earlier trials. A repeated measures ANOVA with mask status (on/off) and time (1st half, 2nd half) demonstrated a main effect of mask status [Exp 1: *F*_(1,65)_ = 20.293, *p* < 0.01; Exp 2: *F*_(1,65)_ = 11.09 *p* < 0.01], but no effect of time nor an interaction between time and mask status(Exp 1: Fs < 1; Exp2: main effect: *F*(1,65) = 2.288, *p* = 0.135. Interaction: *F* < 1). Hence, these analyses suggest that the mask effect was not modulated as a function of experience.

### Experiment 3

The results of Experiments 1 and 2 demonstrated that face recognition abilities are reduced when participants are asked to differentiate between either masked or unmasked faces when wearing a mask themselves. Together, our findings confirm the association between face recognition and observer’s status. However, an alternative hypothesis is that mask-wearing has a more general effect on visual processing, which is not specific to facial recognition and therefore is not related to the observer’s state.

Alterations of the respiration rhythm while wearing a mask could provide a plausible mechanism for such a general decrement in visual processing. It was recently demonstrated that respiration significantly modulates neural oscillations in posterior brain regions as well as the perceptual sensitivity in a near-threshold spatial detection task (Kluger et al., 2021). Mask wearing may modulate some aspects of the respiration process (Fikenzer et al., 2020; Reychler et al., 2021). Accordingly, in a recent study, Fischer and colleagues (2021) utilized transcranial hybrid near-infrared spectroscopies to show that wearing a face mask leads to small, but statistically significant changes in the cerebral hemodynamic and oxygenation of healthy young adults at rest. This effect was still evident for a relatively short period of mask usage.

Thus, Experiment 3 was designed to examine the specificity of the mask-wearing effect on face perception. To this end, participants completed a task that resembled the GFMT requirements, but utilized objects instead of faces as the stimuli. We predicted that no change between mask conditions would be observed in contrast to previous experiments.

### Methods

#### Participants

Eighty participants (40 females, age: 27.8) were recruited online (https://www.prolific.co/) during the month of February 2022, none of whom participated in the previous experiments. Participants were once again compensated for their time (~ $3 CAD for 15 min). All other aspects of the recruitment process were consistent with those used for Experiments 1 and 2. Two participants were excluded from the analysis as their d’ scores were lower than 1. Additionally, data from participants (*n* = 5) who self-reported that they did not wear or remove their masks as instructed was excluded. Hence, the final sample included 73 participants. The reported results are fully replicable when all participants were included in the analysis.

#### Materials

A same-different task was created using novel 3D objects (Freud et al., 2018; Norman et al., 2008). (This task consisted of 164 trials to replicate the GFMT’s structure as closely as possible. The “same” trials consisted of identical image pairs in which one object was rotated 10 degrees on its y-axis and darkened by various shades. Notably, this was done to equate the properties of this particular task to that of the GFMT, in which the “same” trials contain two different images (that differ in brightness and quality) of the same person. The “different” trials consisted of two distinct objects (see Fig. 1 for an example image pair). Participants were again asked to supply their own medical or surgical face mask to wear during the experiment.

#### Procedure

Participants were asked to differentiate between object pairs instead of faces. All other aspects were similar to Experiments 1 and 2.

## Results

**Fig. 4:**
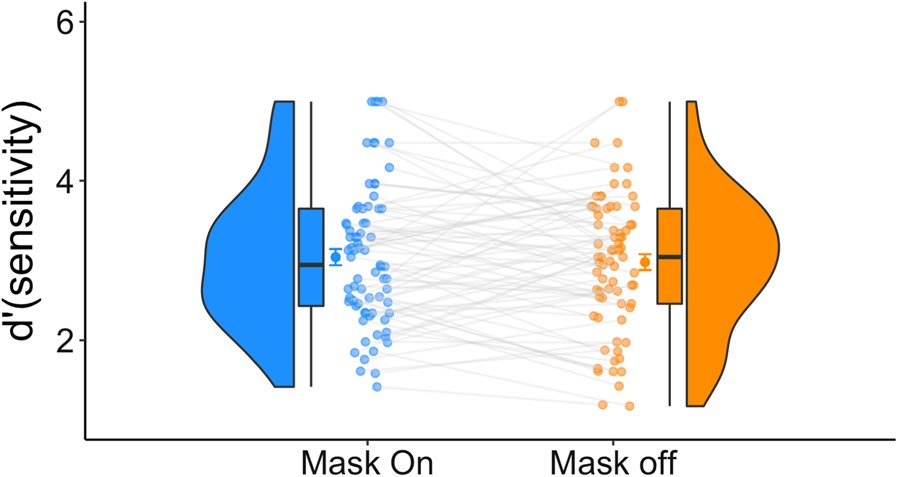
Experiment 3 results. Similar accuracy results were observed for the two masking conditions. For each condition we present the distribution, the individual datapoints, and the quartiles of the dataset. The whiskers depict the rest of the distribution excluding outliers. The darker dots denote the means, and the error bars denote one standard error

Experiment 3 assessed the extent to which wearing a mask interferes with the observers’ ability to recognize objects. With that, participants completed an experiment identical in structure to the GFMT used in Experiments 1 and 2 but featuring 3D objects as the stimuli. A Bayesian t test was conducted and provided moderate evidence in support of the null hypothesis, that is, there is no difference between the two mask conditions [${\text{BF}}_{10}$ = 0.158, Fig. 4]. More specifically, our results showed that $H_{0}$ was 6.3 times more likely than $H_{1}$. Similarity, no effect of mask status on RT was found [${\text{BF}}_{10}$ = 0.14]. In contrast to the findings of Experiments 1 and 2, wearing a mask appears to have no effect on perceptual abilities for non-face stimuli, demonstrating the specificity of the mask-wearing effect. These results weaken the alternative hypothesis, according to which mask-wearing leads to general deficits in visual performance that could be attributed to respiration, a general attentional deficit or occlusion of features in the lower visual field. Instead, this finding reinforces the notion that the observers’ state mediates the deficit observed for face processing.

### Experiment 4

The results of Experiments 1 and 2 showed a robust decrease in face recognition abilities for masked or unmasked faces when participants were asked to wear a mask themselves. At the same time, Experiment 3 demonstrated the specificity of this effect to face perception. Next, we asked whether mask-wearing hinders face perception because the mask itself is positioned on distinctive features of the face (i.e., mouth and nose) or alternatively because participants had to wear an item on their face.

The main hypothesis was that wearing the mask on non-distinctive parts of the face would not hinder face perception abilities. To test this hypothesis, we asked participants to wear their mask in a more unconventional manner than that which they are typically accustomed to (i.e., on their forehead) during select parts of the experiment (see Fig. 1 for an example). Using this approach, the mask covered the same amount of facial area, without occluding the most distinctive features of the face.

### Methods

#### Participants

Eighty participants (40 females, age: 25.2) were recruited online (https://www.prolific.co/) during the month of March 2022, none of whom participated in the previous experiments. Participants were once again compensated for their time (~ $3 CAD for 15 min). All other aspects of the recruitment process were consistent with those used in the experiments described above. Three participants were excluded from the analysis as their d’ scores were lower than 1. Additionally, data from participants (*n* = 7) who self-reported that they did not wear or remove their masks as instructed was excluded. Hence, the final sample included 70 participants. The reported results are fully replicable when all participants were included in the analysis.

#### Materials

The original, extended version of the GFMT was used to assess face perception abilities, and participants were again asked to supply their own medical or surgical face mask to wear during the experiment. An image of one of the researchers wearing a mask on their forehead was included as a demonstration for how participants should be wearing their mask (see Fig. 1 for an example).

#### Procedure

The procedure was the same as that described in previous experiments; however, an additional screening page was included immediately following the mask-wearing instruction page and asked participants to verify that they were wearing their mask before proceeding.

## Results

**Fig. 5:**
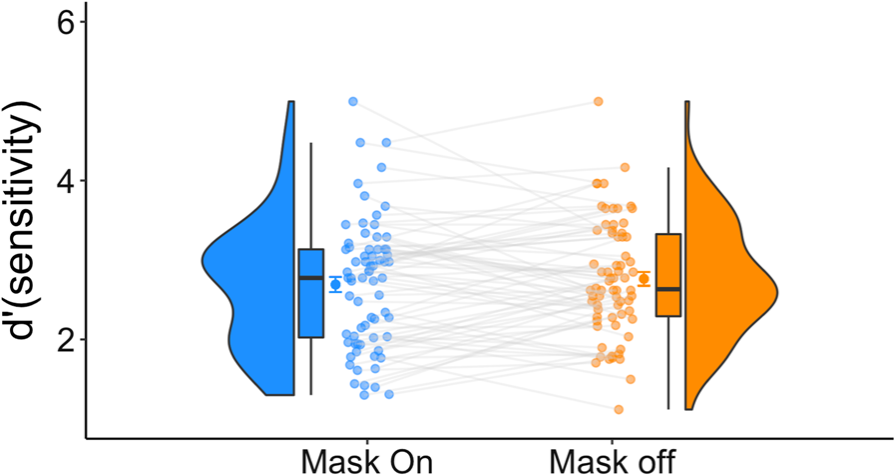
Experiment 4 results. Similar accuracy results were observed for the two masking conditions (mask off and mask on the forehead). For each condition we present the distribution, the individual datapoints, and the quartiles of the dataset. The whiskers depict the rest of the distribution excluding outliers. The darker dots denote the means, and the error bars denote one standard error

Experiment 4 sought to investigate whether the presence of a mask, regardless of its placement, is enough to disrupt face perception. We had participants complete the original, unedited version of the GFMT used in Experiment 1 while wearing a mask on their forehead during either the first or second half of the experiment. The results of a Bayesian paired samples *t* test provide moderate evidence for the null hypothesis (i.e., no effect of masking status), such that the observed data are 5 times more likely under $H_{0}$ than under $H_{1}$ [${\text{BF}}_{10}$ = 0.21] (Fig. 5). A similar pattern was observed in terms of RT [${\text{BF}}_{10}$ = 0.4; the observed data are 2.4 times more likely under $H_{0}$].

### Between experiments comparison

Finally, we directly compared between the four experiments described above. To this end, we employed a repeated measures ANOVA on the accuracy scores with mask status (on, off) and experiment (Experiment 1–4) as independent variables. The Bartlett's test validated the homogeneity of variances (*p* = 0.14). The analysis revealed a main effect of mask status [*F*_(1,271_) = 12.356, *p* < 0.001, *η*_*p*_^2^ = 0.044] (no mask > mask), as well as a main effect of experiment [*F*_(3,271)_ = 12.368, *p* < 0.001, *η*_*p*_^2^ = 0.12] (due to the lower d’ for the masked GFMT—Experiment 2). Importantly, these two main effects were qualified by a two-way interaction between mask status and experiment [*F*_(3,271)_ = 4.513, *p* = 0.004, *η*_*p*_^2^ = 0.048], such that mask status modulates performance only for face stimuli (see OSF repository for the full JASP statistical report). Table 2 includes the descriptive statistics across the different experiments.

**Table 2:** Descriptive statistics for experiments 1–4

Mask status	Group	Mean	SD	*N*
Mask	Experiment 1 (GFMT)	2.581	0.819	66
	Experiment 2 (GFMT Masked)	2.189	0.569	66
	Experiment 3 (Objects)	3.046	0.873	73
	Experiment 4 (Forehead)	2.691	0.807	70
No Mask	Experiment 1 (GFMT)	2.885	0.745	66
	Experiment 2 (GFMT Masked)	2.430	0.586	66
	Experiment 3 (Objects)	2.983	0.855	73
	Experiment 4 (Forehead)	2.763	0.726	70

## Discussion

The unprecedented times of the COVID-19 pandemic dramatically increased the prevalence of mask-wearing across different countries and societies. Consequently, a host of studies aimed to explore the effect of masks on different aspects of face processing—including identity recognition (Carragher & Hancock, 2020; Freud et al., 2020, 2022), emotion recognition (Carbon & Serrano, 2021; Marini et al., 2021) and perceived attractiveness (Parada-Fernández et al., 2022; Pazhoohi & Kingstone, 2022). In the current study, we sought to investigate a novel, and to the best of our knowledge, unexplored attribute of face masks, namely the effect of mask-wearing on face perception. This question also pertains to a more general topic of the association between observers’ states in regard to their body and to other agents and their perceptual behaviors.

In a series of experiments, we have documented substantial changes in face processing abilities when observers were masked. In Experiment 1, we found that mask-wearing decreases face perception abilities for unmasked faces as measured by the GFMT. Experiment 2 replicated and extended these findings by showing that a similar decrease (~ 10%) is also evident when the face stimuli are masked. Hence, the reduced performance in Experiment 1 could not be attributed to the incongruency between the observer’s face and the stimuli.

To test whether mask-wearing has a more general effect on visual processing, we conducted a third experiment using an object recognition task with a similar design to the GFMT. Experiment 3 revealed that mask-wearing has no effect on perceptual abilities for non-face stimuli, as similar accuracy results were obtained across both mask conditions, thereby demonstrating the specificity of the mask-wearing effect. Finally, Experiment 4 sought to further elucidate the observed mask effect and asked whether wearing a mask on non-distinctive parts of the face would likewise hinder face perception abilities. Our results demonstrated that this was not the case, and that the mask effect is contingent on one’s mask being positioned over their nose and mouth.

Previous research demonstrated the association between observers’ bodily states and their perception across different tasks, domains, and modalities. Particularly, manipulation of body posture or hand posture was found to modulate tactile perception (Longo, 2017) and visual perception of size (Kim et al., 2021), letters (Barnett-Cowan et al., 2015) and faces (Barnett-Cowan et al., 2015; Davidenko & Flusberg, 2012). In the domain of face perception, previous research has focused on the effect of somatosensory stimulation (via enforced face pose or brain stimulation) on emotion recognition (Borgomaneri et al., 2020; Niedenthal, 2007; Oberman et al., 2007; Quettier et al., 2021). Across these studies, a reduced emotion recognition ability was identified when participants could not mimic facial expression, or when a TMS was delivered to their somatosensory cortex (Pitcher et al., 2008).

Interestingly, some of the above-mentioned studies (e.g., Borgomaneri et al., 2020; Pitcher et al., 2008) indicated that the effect was specific for emotion recognition, while identity or gender recognition remained intact. In contrast, we found that mask-wearing hinders identity recognition. This discrepancy might be explained by the nature of manipulation. Specifically, previous studies have utilized TMS stimulation, which is relatively localized, and transient in nature. Other studies block facial mimicry by asking participants to hold an item in their mouth, constraining the mobility of the specific muscles around the mouth. In comparison to these manipulations, mask-wearing might generate a more robust, and consistent, somatosensory stimulation across multiple face features (e.g., mouth, nose), which are relevant for face recognition. Notably, masks might not be unique in their effect on face processing. Other clothing items that occlude the lower part of the face (e.g., scarfs) might lead to a similar deficit in face perception abilities.

While mask-wearing modulates the bodily state of observers, it also alters the status of the observers in relation to other agents. Specifically, by adopting the perspective of other agents (i.e., the presented faces), masked observers might consider that their faces are less recognizable, leading to reduced face processing abilities. This involuntary perspective-taking process was termed as altercentric intrusion (Samson et al., 2010). These intrusions were found to influence different cognitive mechanisms including attention, action, perception and memory (for a review see Kampis & Southgate, 2020).

Importantly, the current experiments allowed us to establish the specificity of the mask-wearing effect to face processing. This specificity suggests that the mask-wearing effect is unlikely to be attributed to more general mechanisms such as respiration rate, occlusions to the lower visual field or attention. Nevertheless, the current experimental setting does not allow us to discern the relative contribution of bodily state versus altercentric intrusion to the observed impairment in face perception abilities. As mentioned above, somatosensory stimulation (via TMS or preventing of facial mimicry) was found to influence emotional face processing, but not face recognition per-se (Pitcher et al., 2008; Sel et al., 2014). Hence, one might conclude that the observed mask effect is more likely to be attributed to altercentric intrusion. Future studies should further explore this question.

One important limitation of the current studies is that all data were collected online. While this approach allowed us to collect a large sample size from diverse backgrounds and age ranges, we could not verify participants’ compliance with mask-wearing instructions. We attempted to address this concern by including the post-experiment compliance questionnaire and by analyzing the data only from participants who reported high compliance with the mask-wearing requirement. An additional limitation could be related to demand characteristics of the mask wearing manipulation, particularly given the within-subject design (i.e., each participant was tested with and without a mask and therefore might have assumed the goal of the experiment). Notably, however, Experiment 3 (as well as Experiment 4) weakens this alternative hypothesis, as we utilized a similar within-subject design, and no effect of mask-wearing was observed.

To conclude, the COVID-19 pandemic created a new realm of facial recognition, one in which familiar and unfamiliar faces alike are partially obscured from view. While previous research explored how masks hinder different aspects of face recognition abilities, we demonstrated that mask wearing could also modulate face recognition of unfamiliar, masked and non-masked faces. These findings further establish the association between observers’ bodily states and their visual behaviors.

## Data Availability

Data and analysis code are available on the Open-Source Framework ((Kluger et al., 2021)) under CC-By Attribution 4.0 International license.
